# Oral lesions among HIV seropositive individuals in an era of generic HAART: markers of HAART efficacy?

**DOI:** 10.1186/1471-2334-12-S1-P39

**Published:** 2012-05-04

**Authors:** Janani Vasudevan, Vaishnavi Sivasankar, Umadevi K Rao, Nagalingeswaran Kumarasamy, Kannan Ranganathan

**Affiliations:** 1 Department of Oral and Maxillofacial Pathology, Ragas Dental College, Chennai, India; 2 YRG Centre for AIDS Research and Education, Chennai, India

## Background

Oral lesions have been established as a clinical feature of human immunodeficiency virus (HIV) seropositivity. Treatment with Highly Active Antiretroviral Therapy (HAART) has changed the course of infections with HIV. This study correlates the oral lesion prevalence in HAART and non-HAART groups in a cohort of HIV seropositive patients in Southern India.

## Methods

The study group consisted of 3485 HIV seropositive patients who reported to the dental clinic of YRG CARE, Chennai, India. Oral lesions were diagnosed clinically based on EC Clearinghouse criteria and the data was analyzed using SPSS software.

## Results

In the study population, 92.3% of individuals had acquired the infection through heterosexual contact. The mean age of infected males and females was 35±8 and 30±8 years respectively and the mean CD4 count in males and females was 385.53±226.76 and 284.36±278.28 cells/µL respectively (p<0.05). Patients having CD4 count < 200 (n=908) had more number of oral lesions than patients with CD4 count > 200 (p<0.01). Patients on HAART (n=1042) had a lesser number of lesion than patients not on HAART (p<0.01). Of the lesions, candidiasis was predominantly seen in the non-HAART group (19.7%) as compared to the HAART group (11.7%). Patients who were not on HAART showed a higher prevalence of pseudomembranous candidiasis (12.8% vs.7.9%, p<0.01) and angular cheilitis (6.0% vs.3.7%, p<0.01).

## Conclusion

Patients on Highly Active Antiretroviral Therapy (HAART) demonstrate fewer oral lesions, which can serve as markers of treatment efficacy. Long term follow-up studies are necessary to validate these results.

